# Authors’ Response to Peer Reviews of “Real-World Performance of COVID-19 Antigen Tests: Predictive Modeling and Laboratory-Based Validation”

**DOI:** 10.2196/83474

**Published:** 2025-10-06

**Authors:** Miguel Bosch, Dawlyn Garcia, Lindsey Rudtner, Nol Salcedo, Raul Colmenares, Sina Hoche, Jose Arocha, Daniella Hall, Adriana Moreno, Irene Bosch

**Affiliations:** 1 Info Analytics Innovations, Houston, TX, United States; 2 IDX20 Inc, 166 Clinton Rd, Brookline, MA, 02445, United States, 1 6177347745

**Keywords:** COVID-19, antigen test clinical performance, real-world data, limit of detection lateral flow test, probability of positive agreement, logistic regression

*This is the authors’ response to peer-review reports for “Real-World Performance of COVID-19 Antigen Tests: Predictive Modeling and Laboratory-Based Validation.”*

## Round 1 Review

We thank the reviewers for the thoughtful feedback on our paper [1]. Below, we address each of their points.

### Reviewer BH [2]

#### Minor Comments

*1. It would be good to include a schematic/analysis/methods and an image of how the lateral flow assays look and how test band intensities are obtained. Was that done with ImageJ?*

**Response:** We agree with the suggestion. The request has been addressed by including Figure 1B in the revised manuscript. The figure legend has also been updated and matches the new Figures 1A and 1B.

The software used is like ImageJ, but we did not use ImageJ. ImageJ was used in prior work [3]. The methodology we present here used a newly made software. The Python and R scripts that were utilized in this software have been posted on a website and are available to the public. The new References section includes the website.

*2. An interesting aspect of the paper is the comparison between trained eye versus user of lateral flow assays (Figure 5). I think that adding a paragraph about the conclusions from that figure might be good in the Discussion section.*

**Response:** The new version of the manuscript includes a paragraph in the Discussion section that explains the finding of trained eye (study staff) versus community users. Another paragraph was added to the Methods section explaining the training given to all community participants to properly report the test data.

### Reviewer FZ [4]

#### Major Comments

*1. The authors’ clinical conclusions based on their prediction theory are overly optimistic.*

**Response:** We agree to tone down our optimism. We modified the Abstract following this concern. We included in the new version of the manuscript cautionary notes and listed points of consideration.

*2. The authors can explore actual clinical evaluations to determine the robustness of their prediction modeling.*

**Response:** We agree with the follow-up plan suggested. We have addressed this concern by submitting a separate manuscript, currently “in press” at *JMIR Bioinformatics and Biotechnology*. The preprint of the work that includes the clinical evaluation of multiple test brands is titled “Improving Antigen Test Sensitivity Estimation through Target Distribution Balancing” and currently available here [5].

*3. Thus, the paper merits publication, providing the limitations are more clearly described and the conclusions are limited to the mathematical results for which the authors have proven their claims theoretically. Extension to clinical applicability is a different story yet to be told.*

**Response:** We agree with this comment; as explained in a previous response, we have extended the clinical applicability of these methods, and the data are now in press in a *Journal of Medical Internet Research* sister journal as mentioned before.

*4. The authors should be encouraged to move forward in view of the need and the poor performance of COVID-19 rapid antigen tests during the pandemic because of low sensitivity, a lack of deep understanding, and the “prevalence boundary,” a measure of when the rate of false omissions becomes too high and false negatives spread disease.*

**Response:** The updated reference list indicates we agree with the concern. We introduced two references to illustrate that there are mitigation strategies for less sensitive diagnostic tests via serial testing (ie, testing on consecutive days during the acute COVID-19 disease stage results in an increase in the test sensitivity).

#### Minor Comments

*5. Needs English grammar review. This could be achieved by using an artificial intelligence editor.*

**Response:** We thank the reviewer for the suggestion. We used a grammar artificial intelligence corrector, and we introduced several changes to the original manuscript as a result of this review. A version of the modified manuscript that includes all changes to English grammar errors is available.
